# Retraction: Sub-lethal doses of Nucleopolyhedrosis Virus and synthetic ınsecticides alter the biological parameters of *Helicoverpa armigera* Hübner (Lepidoptera: Noctuidae)

**DOI:** 10.1371/journal.pone.0272187

**Published:** 2022-08-03

**Authors:** The PLOS ONE Editors

The *PLOS ONE* Editors retract this article [1] because it was identified as one of a series of submissions for which we have concerns about authorship, competing interests, and peer review. We regret that the issues were not addressed prior to the article’s publication.

MSS either did not respond directly or could not be reached. ADA, SMZ, SS, NI and MNN did not agree with the retraction.
